# Physical and Mechanical Therapies for Lower Limb Problems in Children With Juvenile Idiopathic Arthritis: A Systematic Review With Meta‐Analysis

**DOI:** 10.1002/jfa2.70096

**Published:** 2025-11-04

**Authors:** Antoni Fellas, Fiona Hawke, Mohammed Maarj, Davinder Singh‐Grewal, Derek Santos, Andrea Coda

**Affiliations:** ^1^ School of Health Sciences College of Health Medicine and Wellbeing The University of Newcastle Newcastle Australia; ^2^ Equity in Health and Wellbeing Research Program Hunter Medical Research Institute (HMRI) Newcastle Australia; ^3^ Sydney Children's Hospital Network Westmead Australia; ^4^ Sydney Children's Hospital Network Randwick Australia; ^5^ School of Health Sciences Queen Margaret University Edinburgh UK

**Keywords:** foot and ankle, juvenile idiopathic arthritis, lower limb

## Abstract

**Objective:**

To systematically review the evidence for physical (for e.g. strengthening) and mechanical (for e.g. foot orthoses) therapies for lower limb problems in children with juvenile idiopathic arthritis (JIA).

**Methods:**

Randomised clinical trials of physical and mechanical interventions for lower limb problems in children with JIA were included. The primary outcome was pain and secondary outcomes included disability, functional ability, and quality of life. Electronic databases were searched for eligible studies. Authors of included studies and researchers in the field were contacted to identify additional studies.

**Results:**

Two authors independently screened 4876 titles and abstracts. Eleven randomised clinical trials were ultimately included. Four studies explored the effect of foot orthoses and seven physical therapies. Studies evaluating the effect of foot orthoses on foot and ankle pain were pooled in a meta‐analysis. Results showed that foot orthoses were statistically and clinically significant in reducing both parent (−11.08 [−20.25, −1.90]) and child (−21.45 [−30.18, −12.73]) reported foot and ankle pain after 3 months compared to the control. This significant effect was sustained post 3 months. Physical therapies such as Pilates and underwater exercises were statistically and clinically significant in reducing lower limb pain after 3 months of intervention.

**Conclusion:**

Foot orthoses can be prescribed to significantly reduce foot and ankle pain for children with JIA. Physical therapies appear to reduce pain during 3 months of intervention versus the control but are currently hampered by lack of blinding. Further research is also required to determine the effect of physical therapies past 3 months.

**Abbreviations**
CIs: confidence intervals
JIA: juvenile idiopathic arthritis
JRA: juvenile rheumatoid arthritis
PedsQL: Paediatric quality of life inventory
RCTs: randomised clinical trials
VAS: visual analogue scale

## 1 Introduction

Juvenile idiopathic arthritis (JIA) is the most prevalent rheumatic disease among children [1]. Despite advancements in medical treatments, numerous children with JIA face physical challenges such as decreased participation in physical activities [2], difficulties in attending school [3], and psychological issues [4]. The primary goal in managing JIA is to achieve disease remission [5]. Achieving remission depends on various factors, including early diagnosis followed by timely and targeted interventions [5]. The subtype of JIA also influences the likelihood of active synovitis and the overall prognosis [1]. For instance, while lower limb pathology is present in approximately 50% of JIA cases, extra‐articular manifestations are notably common in children with the enthesitis‐related subtype [6]. A 5‐year prospective study conducted in 2018 on children with JIA found lower limb synovitis and walking disabilities were prevalent at initial presentation [7]. Specifically, knee joint synovitis was observed in 71% of the cohort, and ankle synovitis in 34% at baseline. However, after 5 years, around 25% of the participants continued to experience walking difficulties despite a significant reduction in clinically assessed lower limb joint disease [7]. Recent studies have also underscored the impact of JIA on gait characteristics and walking function, even with advancements in pharmacological care [8, 9, 10]. Therefore, incorporating non‐pharmacological therapies could play a crucial role in addressing secondary functional issues in children and adolescents with JIA [11].

In 2017, we published a systematic review with meta‐analysis to evaluate the evidence for physical and mechanical therapies specifically targeting lower limb issues in children and adolescents with JIA [12]. The search, conducted in 2015, identified only two randomised clinical trials (RCTs) aimed at reducing pain and/or improving functional outcomes in the lower limb [12]. Both studies investigated the effects of custom or customised foot orthoses on reducing foot and ankle pain in their respective JIA cohorts [13, 14]. The review found non‐statistically significant reductions in the primary outcome of pain when data were combined in a meta‐analysis [12]. At the time of the search, no physical therapy RCTs had explored the effects of reducing symptoms and improving lower limb function [12], despite non‐pharmacological care, including physiotherapy, occupational therapy, and podiatry, being recommended for children with JIA in national guidelines [15, 16, 17]. However, many of these recommendations are outdated or limited by low‐quality supporting evidence. Therefore, this systematic review aims to provide an updated assessment of the effects of physical and/or mechanical therapies in managing lower limb problems in children and adolescents with JIA.

## 2 Methods

The original protocol was published elsewhere [18] and registered in PROSPERO (https://www.crd.york.ac.uk/prospero/display_record.php?ID=CRD42023443178).

### 2.1 Studies and Participants

All RCTs and quasi‐RCTs examining mechanical and physical interventions for lower‐limb issues in JIA were included. Physical interventions encompassed activities such as stretching, strengthening, hydrotherapy, and massage. Mechanical interventions included foot orthoses, footwear, and splints. Studies focusing on acupuncture, pharmacological, and surgical therapies were excluded, as were those evaluating interventions aimed at preventing lower‐limb problems in JIA. Eligible studies included children diagnosed with JIA and experiencing one or more lower‐limb issues. A lower‐limb problem was defined as any pathological condition (e.g., pain, muscle atrophy, flexion contracture) affecting the lower limb, from the trunk to the glutaeal region, femoral region, knee, leg, ankle, and foot. Studies conducted in any setting, such as public and community health services, private clinics, preschools, and schools, were included.

### 2.2 Outcomes

The primary outcome was any validated quantifiable measure of pain, such as the Visual Analogue Scale (VAS) or Paediatric Pain Questionnaire [19]. Secondary outcomes included disability (e.g., Juvenile Arthritis Foot Disability Index [20]), functional ability, health‐related quality of life (HRQoL) (e.g., the Paediatric Quality of Life Inventory [PedsQL] measurement model) [21], participant satisfaction with the intervention, and adverse events.

### 2.3 Search Strategy

This systematic review updates the search conducted in January 2015 [12]. The MEDLINE search strategy (January 2015 to February 2025) is detailed in Table 1. This strategy was adapted for Embase (January 2015 to February 2025), Cochrane Central Register of Controlled Trials (The Cochrane Library, latest issue), PubMed (January 2015 to February 2025), and Cumulative Index to Nursing and Allied Health Literature (January 2015 to February 2025). No language or publication restrictions were applied. Reference lists of all included studies were reviewed for additional eligible trials. Two reviewers (A.F. and A.C.) independently screened the titles and abstracts of all identified studies. Full‐text articles of potentially eligible studies were retrieved by A.F. and independently screened by A.F. and A.C. Authorship and results were not masked. Disagreements regarding full‐text inclusion were resolved by a third reviewer (F.H.). If disagreements persisted, study authors were contacted, though this was not required.

**TABLE 1:** OvidSP MEDLINE search strategy.

1.	Randomised controlled trial.pt.
2.	Controlled clinical trial.pt.
3.	randomised.ab.
4.	randomised.ab.
5.	placebo.ab.
6.	randomly.ab.
7.	trial.ab.
8.	groups.ab.
9.	1 or 2 or 3 or 4 or 5 or 6 or 7 or 8
10.	Exp animals/not humans.sh
11.	9 not 10
12.	Juvenile idiopathic arthritis
13.	Juvenile chronic arthritis
14.	Juvenile rheumatoid arthritis
15.	JIA
16.	12 or 13 or 14 or 15
17.	11 and 16

Abbreviations: ab: abstract; JIA: juvenile idiopathic arthritis; pt: publication type; sh: subject heading.

A.F. extracted data from included studies using a standardised pilot‐tested form, and a second author (A.C.) checked all extracted data. Study authors were contacted for any missing or unclear information. Inconsistencies in data extraction were discussed between A.F. and A.C., with arbitration by F.H. if necessary.

### 2.4 Risk of Bias

Risk of bias for each included study was independently assessed by F.H. and M.M. using criteria from the Cochrane Handbook for Systematic Reviews of Interventions [22]: (1) sequence generation; (2) allocation concealment; (3) blinding of participants, personnel, and outcome assessors; (4) incomplete outcome data; (5) selective outcome reporting; and (6) other sources of bias. Each criterion was rated as high, low, or unclear risk of bias. Disagreements were resolved by discussion between F.H. and M.M.

### 2.5 Statistical Analysis

Group means and standard deviations were analysed in Review Manager [23] to produce mean differences and 95% confidence intervals (CIs). Standardised mean difference analyses were conducted when different measurement scales were used in a single meta‐analysis. We planned to pool different follow‐up periods after adjustment if steady rates of change could be demonstrated, however, this was not necessary.

### 2.6 Assessment of Heterogeneity

Different types of interventions (e.g., foot orthoses vs. splints) and different types of lower limb problems (e.g., foot pain vs. gait instability) were analysed separately.

Clinically homogeneous studies in terms of outcomes, participants, and interventions were pooled in meta‐analysis. Intertrial statistical inconsistency was quantified using I2, calculated as follows: I2 = 100% [(Q–df)/Q], where Q is Cochran's heterogeneity, v2 statistic, and df represents degrees of freedom. Cochran's Q was obtained by summing the squared deviations of each trial's estimate from the overall meta‐analytic estimate, with a *p* value compared to a v2 distribution with k–1 degrees of freedom (where k is the number of trials). I2 values were interpreted as follows: 0%–40% might not be important, 30%–60% may represent moderate heterogeneity, 50%–90% may represent substantial heterogeneity, and 75%–100% may represent considerable heterogeneity [22]. A random‐effects model was used to incorporate heterogeneous trials in a meta‐analysis when heterogeneity could not be explained.

### 2.7 Data Synthesis

A.F. constructed data analyses in The Cochrane Collaboration's statistical package Review Manager 5. A.F. then entered the data, which were checked by A.C. for accuracy.

### 2.8 Subgroup Analyses

No subgroup analyses were planned as different types of interventions and different presentations of lower limb problems were analysed separately in the primary analyses.

### 2.9 Sensitivity Analysis

Sensitivity analyses were performed by excluding trials that failed to blind participants or conceal allocation. If outliers contributed to heterogeneity and the reason for the discrepancy was evident, analyses with and without the outlying trials were performed.

## 3 Results

### 3.1 Studies

Overall, 5937 titles and abstracts were retrieved from the search in which 1061 were automatically removed as duplicates. 4876 studies were screened independently, and 22 studies were identified as being potentially eligible. Of the 22 potentially eligible studies, 11 were excluded: five studies were the wrong study design [24, 25, 26, 27, 28]; two were the wrong intervention [29, 30]; one was not specific to the lower limb [31]; and one was a protocol paper only [32]. Finally, a further two studies were excluded as authors confirmed via email they were unable to provide follow‐up data for the trial and control groups [33, 34].

**FIGURE 1:**
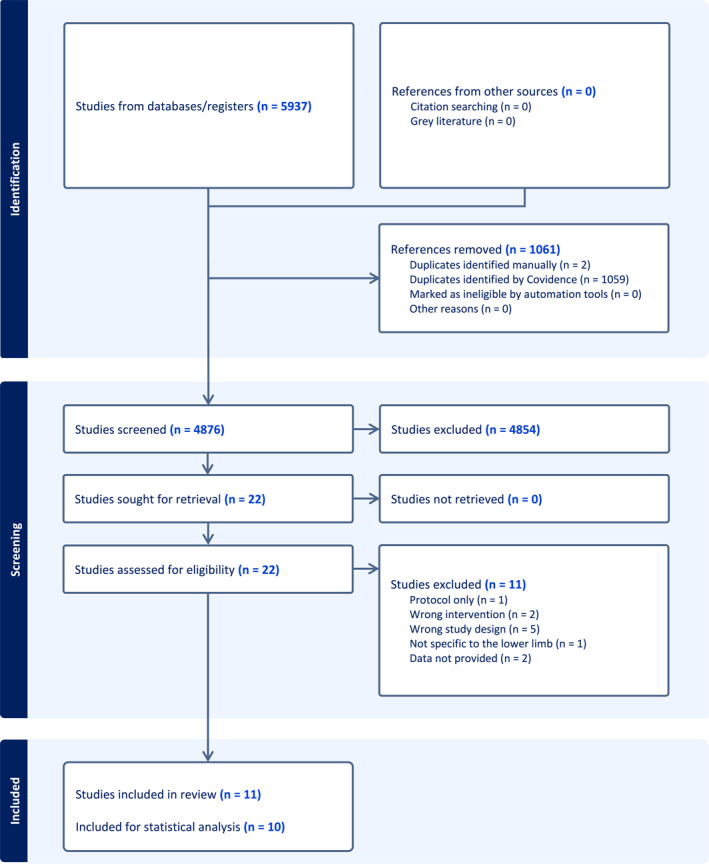
PRISMA flow diagram generated from Covidence software.

Overall, 11 studies were included in this review [13, 14, 35, 36, 37, 38, 39, 40, 41, 42, 43], one of which was unable to be included in Review Manager 5 statistical analyses as authors of that study did not provide parametric (mean and standard deviation) data [35]. Figure 1 depicts the PRISMA flow diagram. Four studies explored the effect of custom or customised foot orthoses [13, 14, 39, 42]; three studies tested different exercises programs including Pilates, balance‐proprioceptive and core stability [35, 38, 40]; two studies explored the effect of a water‐based intervention [37, 41]; one study explored the effect of electromyographic biofeedback training [36]; and one accommodating variable resistance training [43].

### 3.2 Participants

A total of 510 participants across 11 studies were included in this review. Ten of the eleven studies included participants diagnosed using the International League of Associations for Rheumatology [44] classification system [13, 14, 35, 37, 38, 39, 40, 41, 42, 43]. One study included participants diagnosed as having juvenile rheumatoid arthritis (JRA) using the American College of Rheumatology criteria [36]. The age range of participants were 5–19 years old. Overall, the studies included a total of 170 boys and 340 girls. One study did not include gender data [37]. Of the 510 participants diagnosed as having JIA or JRA, 32 had enthesitis‐related, 347 had polyarticular, 106 had oligoarticular, 14 had systemic, 9 had psoriatic arthritis and 2 with undifferentiated. There was a high proportion of participants with the polyarticular subtypes (rheumatoid factor positive and negative) due to six of the 11 studies only recruited participants with this subtype [36, 37, 38, 40, 41, 43]. Table 2 tabulates the characteristics of all included studies.

**TABLE 2:** Characteristics of included studies.

Study	Lower limb problem	Intervention groups	Participants (no.)	Age range (years)	Recruitment source	Follow‐up(s)	Outcome(s)
Powell et al. (2005)	Pain in foot and ankle	1. Customs foot orthoses 2. Neoprene inserts. 3. Supportive shoes alone	40	5 to 19	Three Paediatric rheumatology clinics in southern California	3 months	1. Pain: VAS. 2. Function: Foot function index. 3. Ambulation speed: Timed walking test. 4. HRQoL: Generic PedsQL 4.0 scores, physical
Coda et al. (2014)	Pain in foot and ankle	1. Customised foot orthoses. 2. Sham orthoses	60	5 to 18	Two hospitals in Scotland	3 and 6 months	1. Pain: VAS. 2. HRQoL: PedsQL rheumatology scores, generic PedsQL scores, physical functioning scale (unpublished)
Baydogan et al. (2015)	Inflammation and/or movement restrictions in the knee.	1. Exercise program (muscle stretching and strengthening). 2. Exercise + proprioceptive‐balance exercises	30	6 to 18	Department of pediatric rheumatology, Istanbul	3 months	1. Pain: VAS. 2. Passive range of motion (knee flexion and extension). 3. Muscle strength (quadriceps and hamstrings, using handheld portable dynamometer. 4. Static balance, functional reach test. 5. Postural balance control, using flamingo balance test. 6. Quality of life, CHAQ scores. 7. Walking function, 10‐m walking test.
Elnaggar et al. (2016)	Polyarticular JIA with bilateral knee involvement	1. Traditional physical therapy (hot‐packs, range of motion exercises, isometric exercises, weight‐bearing exercises, cycling). 2. Underwater training program for strengthening quadriceps and hamstrings.	30	No age range provided. Average age in all participants 9.9 years	King Khalid hospital, Al‐Kharj, Saudi Arabia	1 and 3 months	1. Quadricep and hamstring muscle strength, peak torque using HUMAC NORM CSMI 2009 USA testing and rehabilitation system. 2. Pain: Visual analogue scale 0–10.
Eid et al. (2016)	Polyarticular JRA knee involvement	1. Standard physical therapy. 2. Standard physical therapy + EMG biofeedback‐guided isometric exercises.	36	8 to 13	Physical therapy department, outpatient clinic, College of applied medical sciences, Najran University, Najran, Saudi Arabia	3 months	1. Pain: VAS, 0–10. 2. Quadricep muscle strength: peak torque using the Biodex system 3 multi‐joint system testing and rehabilitation (Biodex medical system, shirley, NY)
Fellas et al. (2021)	Active involvement of the lower limb (must include at least foot and/or ankle)	1. Customised foot orthoses. 2. Sham orthoses	66	5–18 years	Three outpatient pediatric rheumatology hospital clinics in NSW, Australia	1, 3, 6 and 12 months	1. Pain: VAS, 0–100 mm. 2. Quality of life: PedsQL 3.0 rheumatology module. 3. Foot and ankle disability: Juvenile arthritis foot ankle disability index. 4. Swollen and tender joints.
Elnaggar et al. (2021)	Polyarticular JIA, lower limb involvement	1. Core stability exercises + conventional physical therapy exercises. 2. Conventional physical therapy.	36	10–14 years	Pediatric rheumatology Department, King Khalid hospital, Al‐Kharj, Saudi Arabia.	3 months	1. Bone Densitometry (of femur and lumbar spine). 2. Functional capacity 6‐min walk test.
Fellas et al. (2022)	Active involvement of the lower limb (must include at least foot and/or ankle)	1. Customised foot orthoses. 2. Sham orthoses	66	5–18 years	Three outpatient pediatric rheumatology hospital clinics in NSW, Australia	3 and 6 months	1. Pressure time integrals. 2. Peak pressures. 3. Cadence. 4. Stance time. 5. Swing time
Elnaggar et al. (2022)	Polyarticular JIA with bilateral knee involvement	1. Aqua‐plyometric training. 2. Standard exercise protocol	48	12–18 years	Three large pediatric rheumatology hospital clinics in Riyadh, Saudi Arabia	3 months	1. Muscle strength (Isokinetic dynamometer); 2. Bone Densitometry (of femur and lumbar spine); 3. Physical fitness (6‐min walk test)
Azab et al. (2022)	Polyarticular JIA, lower limb involvement	1. Pilates + conventional physical therapy. 2. Conventional physical therapy	40	10–14 years	Pediatric rheumatology Department, King Khalid hospital, Al‐Kharj, Saudi Arabia.	3 months	1. Pain (VAS); 2. Cardiorespiratory fitness (peak VO2, max heart rate, breath‐by‐breath minute ventilation); 3. Functional ability (CHAQ); 4. Quality of life (PedsQL).
Elnaggar et al. (2024)	Polyarticular JIA with bilateral knee involvement	1. Accommodating variable resistance training. Standard exercise protocol	58	12–18 years	Pediatric rheumatology Department, King Khalid hospital, Al‐Kharj, Saudi Arabia and two other referring hospitals in Riyadh, Saudi Arabia.	6 weeks	1. Muscle architecture; 2. Peak torque; 3. Functional performance (6‐min walk test, timed up and down stairs test, shuttle run test).

Abbreviations: BMI: body mass index; CHAQ: childhood health assessment questionnaire; EMG: electromyographic feedback; HRQoL: health‐related quality of life; JIA: Juvenile idiopathic arthritis; JRA: juvenile rheumatoid arthritis; PedsQL: Paediatric quality of life inventory; VAS: visual analogue scale.

### 3.3 Lower Limb Problems

Four studies required participants to exhibit at least foot and/or ankle pain on recruitment [13, 14, 39, 42]. Four studies recruited participants who had either unilateral or bilateral knee involvement which included inflammation, pain, joint restriction or quadricep muscle weakness [35, 36, 37, 41]. Lastly, two more studies specified participants were recruited if they exhibited involvement in any lower limb joint [38, 40].

### 3.4 Setting

All studies recruited participants from pediatric rheumatology hospital outpatient services. Five of the 10 included studies were multicentre [13, 14, 39, 41, 42] with the remaining studies recruiting participants from a single outpatient clinic [35, 36, 37, 38, 40]. The geographical locations of clinics included: United States of America, United Kingdom, Turkey, Saudi Arabia and Australia.

### 3.5 Types of Physical and Mechanical Therapies

#### 3.5.1 Mechanical Therapies

##### 3.5.1.1 Foot Orthoses

Four studies explored the effect of a mechanical therapy [13, 14, 39, 42]. These studies explored the effect of either custom [14] or customised prefabricated [13, 39, 42] foot orthoses. Custom foot orthoses are devices that are manufactured (typically by an orthoses laboratory) based on an impression of the patient's feet. Impressions can be attained by plaster casting, foam box impression or digital scanning (typically captured with a phone, tablet or 3D Scanner). Customised prefabricated foot orthoses are devices that are already premade in different sizes. These can be available in full or half length, which are then customised by the clinician based on the biomechanical needs of the patient. The intention of these devices is to provide a combination of biomechanical correction, pressure redistribution or offloading and provide better shock absorption in the feet [45]. The custom foot orthoses in Powell et al. (2005) were made from semi‐rigid material (metal particle‐reinforced polyolefin) and casts of participants in the intervention group were taken when the sub‐talar joint was in a neutral position [14]. Coda et al. (2014), Fellas et al. (2021) and Fellas et al. (2022) used customised prefabricated foot orthoses to achieve mechanical support for their respective intervention groups [13, 39, 42]. Customised prefabricated foot orthoses were produced based on a pre‐made generic full‐length device which were then customised chair‐side based on participants biomechanical needs and foot complaints. Customisations may include but limited to adding additional midfoot arch support, deflections off painful and/or swollen plantar joints such as the metatarsophalangeal joints or increasing rearfoot posting or correction (for e.g. rearfoot varus wedge to reduce excessive rearfoot pronation). Powell et al. (2005) also included a third arm in their study which included a group that were allocated a prefabricated flat device made from neoprene that were not modified.

#### 3.5.2 Physical Therapies

Seven studies explored the effect of a physical therapy [35, 36, 37, 38, 40, 41, 43].

##### 3.5.2.1 Proprioceptive‐Balance Training

The first study explored the effect of a proprioceptive‐balance exercise program [35]. Participants in this exploratory group received three supervised sessions a week for 12 weeks which included exercises to improve lower limb strength and balance. These exercises included: stepping back and tandem walking (25m); standing on one foot; knee flexion‐extension on one leg; forward, sideways and backward bending on one leg with eyes open and closed; balance board and mini trampoline exercises [35]. The exploratory group also received bicycle, stretching and strengthening exercises that the control group received.

##### 3.5.2.2 Underwater Resistance Training and Interferential Current Therapy (IFC)

Elnaggar et al. (2016) explored the effect of a combined therapy approach of underwater resistance exercise program and IFC [37]. After a 5‐min warm‐up participants in the exploratory group completed 20 min of resistive underwater exercises for the knee joint (flexion and extension). Once cooled down they then received 15 min of IFC to the knee joint. The knee joints were allocated an amplitude modulated constant frequency of 100 Hz and a pulse duration of 125 µsecs using the using Endomed 682 Platinum Interferential Modular (Enraf‐Nonius, Netherlands). This program was conducted over a 3‐month period, however, authors did not specific the frequency of these sessions in their published paper [37].

##### 3.5.2.3 Electromyographic (EMG) Biofeedback Training

Eid et al. (2016) evaluated the use of EMG biofeedback training on the quadricep muscles [36]. Both groups in this study received a standardised exercise program for the quadricep muscles, while the exploratory group also received a 12‐week, 3 days a week for 15 min each of EMG biofeedback‐guided isometric exercises. Isometric exercises were used as authors did not want to exacerbate any acutely inflamed joints. The Myomed 932 (Enraf Nonius, Rotterdam, The Netherlands) which is a 2‐channel EMG machine with full screen displaying the EMG signal with a curve obtained for both vastus medialis oblique, and rectus femoris was used in this study [36]. Specific details of the electrode placement and procedure are well documented in the original published manuscript [36].

##### 3.5.2.4 Core Stability Exercises

Elnaggar et al. (2021) explored the use of core stability exercises. During the 3‐month observational period, participants in both groups received a conventional physical therapy program three times a week for 30 min per session. This program included general active range of motion exercises, stretching exercises, isometric training and other various activities such as treadmill or bicycle. For those only randomised to the intervention group, participants received the core stability exercise program which included eight different core exercises. The core stability program took 45 min to complete and was performed three times per week for 3 months. Each exercise had 10 or 20 repetitions, depending on if it was conducted per side of the body or in general. Intervals of rest were provided between exercises based on the child's needs and all exercises were performed on an exercise mat on the floor. The full text publication details each exercise and how they were performed [38].

##### 3.5.2.5 Aqua‐Plyometric Exercises

Elnaggar et al. (2022) explored the effect of an aquatic‐based exercise program over a 12‐week observation period. Participants randomised to the exploratory group conducted this program twice weekly with each session taking around 45 min [41]. The training program was conducted in a waist‐deep pool and adopted a gradual increase approach with respect to training load. The session was divided into 3 stages and supervised by the same research therapist: (1) Warm‐up (10 min) including slow jogging in the water; (2) main workout (25–30 min) including 10 different aqua‐plyometric exercises, all of which focused on lower limb joints and involved single and double leg hops and jumps (full program is provided by the authors as a supplementary file); (3) cooldown (5 min of gentle walking).

##### 3.5.2.6 Pilates

One study explored the effect of a Pilates exercise program [40]. The Pilates program was conducted over a 3‐month period and was completed 3 times per week for 25 min each session. The session was supervised by a pediatric physical therapist and included the following exercises: hundred exercise, one leg stretch, hip twist, knee stretches, arm openings, plank, standing footwork, squat, swimming with stabilisation ball and wall squat rolls. Each exercise is detailed in the full text publication. The general prescription was that each exercise consisted of 5 repetitions with 3 sets each [40].

##### 3.5.2.7 Accommodating Variable‐Resistance Training

Elnaggar et al. [43] tested the effect of accommodating variable‐resistance training versus standard exercise program. The variable‐resistance program was designed to strengthen the extensors of the knee and was completed by participants three times per week for 6 weeks. The workout required participants to complete three sets of 5–10 repetitions of maximum‐effort concentric actions at three variable angular speeds (240, 180 and 120°). An interval rest of 2–3 min was allowed between each set.

##### 3.5.2.8 Risk of Bias

Table 3 outlines the results of the risk of bias assessment of all included studies. Almost all studies were able to report a sequence generation; however, allocation concealment was unclear for at least half of the included RCTs. Blinding of participants, personnel and outcome assessors was unsuccessful for almost all the included studies which is a consistent and well reported issue with non‐pharmacological based RCTs [46].

**TABLE 3:** Risk of bias.

	Sequence generation	Allocation concealment	Blinding of participants	Blinding of personnel	Blinding of outcome assessors	Incomplete data outcome	Selective outcome reporting	Other sources of bias
Powel et al. (2005)	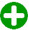	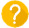	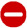	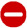	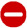	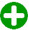	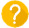	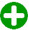
Coda et al. (2014)	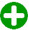	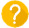	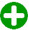	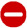	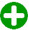	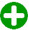	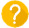	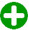
Baydogan et al. (2015)^a^	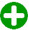	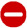	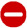	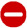	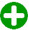 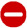 ^a^	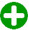	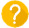	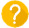
Eid et al. (2016)	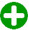	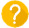	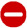	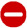	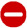	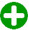	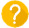	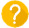
Elnaggar et al. (2016)	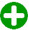	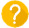	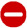	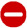	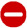	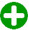	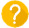	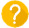
Elnaggar et al. (2021)	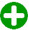	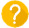	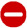	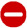	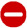	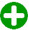	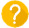	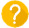
Fellas et al. (2021)	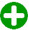	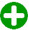	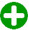	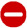	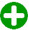	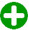	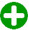	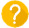
Fellas et al. (2022)	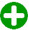	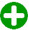	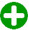	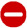	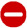	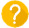	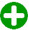	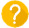
Elnaggar et al. (2022)	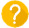	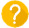	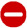	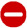	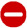	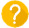	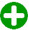	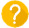
Azab et al. (2022)	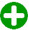	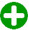	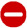	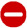	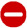	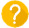	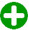	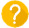
Elnaggar et al. (2024)	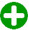	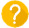	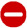	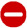	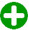	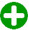	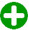	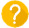

^a^ Research therapists were blinded however participants were not.

### 3.6 Outcomes

The outcomes from each study have been summarised as part of Table 2.

### 3.7 Comparisons

#### 3.7.1 Physical Therapies

The following exploratory group interventions were compared to standard physical therapy alone: electromyographic biofeedback training and standard physical therapy [36]; resistive underwater and interferential current exercise training [37]; core stability exercises and standard physical therapy [38]; Pilates and standard physical therapy [40]; aqua plyometric exercises [41]; and accommodating variable‐resistance training [43].

#### 3.7.2 Mechanical Therapies

Three of the four studies exploring the effect of a mechanical intervention were two‐armed clinical trials in which they compared customised prefabricated foot orthoses to sham foot orthoses [13, 39, 42]. The sham orthoses were flat insoles without any corrective modifications. The fourth study was three‐armed in which authors compared custom‐made foot orthoses to a group receiving flat neoprene inserts to another group receiving new athletics shoes alone. It is worth noting that the custom foot orthoses and neoprene inserts groups also received new athletic shoes [14].

### 3.8 Data and Analysis

**FIGURE 2:**
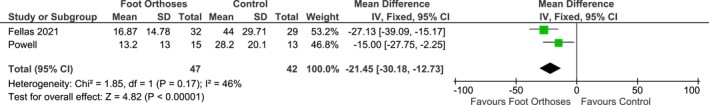
Meta‐analysis of the effectiveness of custom/customized foot orthoses versus a control (sham orthoses or supportive footwear alone) in treating child‐reported foot and ankle pain after 3 months in children with juvenile idiopathic arthritis. CI, confidence interval; IV, inverse variance.

**FIGURE 3:**
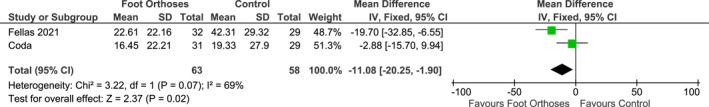
Meta‐analysis of the effectiveness of customised prefabricated foot orthoses versus a control (sham orthoses) in treating parent‐reported foot and ankle pain after 3 months in children with juvenile idiopathic arthritis. CI, confidence interval; IV, inverse variance.

**FIGURE 4:**
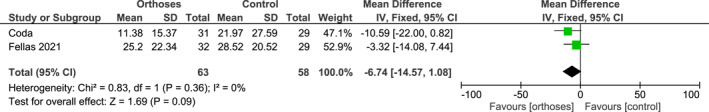
Meta‐analysis of the effectiveness of customised prefabricated foot orthoses versus a control (sham orthoses) in treating parent‐reported foot and ankle pain after 6 months in children with juvenile idiopathic arthritis. CI, confidence interval; IV, inverse variance.

**FIGURE 5:**
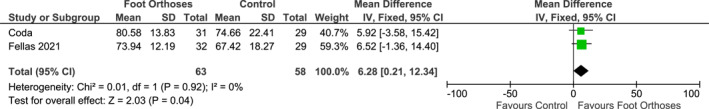
Meta‐analysis of the effectiveness of customised prefabricated foot orthoses versus a control (sham orthoses) in improving health‐related quality of life after 3 months in children with juvenile idiopathic arthritis. This figure represents the child‐reported scores of the Paediatric Rheumatology, Paediatric Quality of Life Inventory. CI, confidence interval; IV, inverse variance.

**FIGURE 6:**
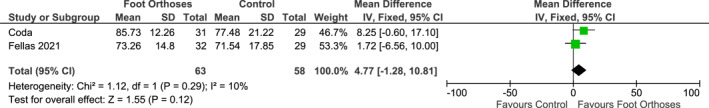
Meta‐analysis of the effectiveness of customised prefabricated foot orthoses versus a control (sham orthoses) in improving health‐related quality of life after 6 months in children with juvenile idiopathic arthritis. This figure represents the child‐reported scores of the Paediatric Rheumatology, Paediatric Quality of Life Inventory. CI, confidence interval; IV, inverse variance.

**FIGURE 7:**
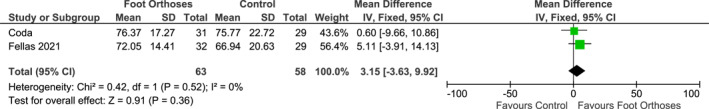
Meta‐analysis of the effectiveness of customised prefabricated foot orthoses versus a control (sham orthoses) in improving health‐related quality of life after 3 months in children with juvenile idiopathic arthritis. This figure represents the parent‐reported scores of the Paediatric Rheumatology, Paediatric Quality of Life Inventory. CI, confidence interval; IV, inverse variance.

**FIGURE 8:**
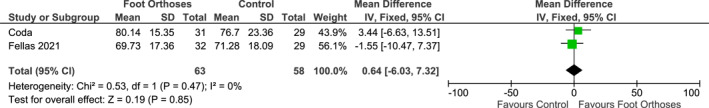
Meta‐analysis of the effectiveness of customised prefabricated foot orthoses versus a control (sham orthoses) in improving health‐related quality of life after 6 months in children with juvenile idiopathic arthritis. This figure represents the parent‐reported scores of the Paediatric Rheumatology, Paediatric Quality of Life Inventory. CI, confidence interval; IV, inverse variance.

**FIGURE 9:**
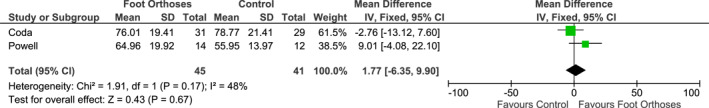
Meta‐analysis of the effectiveness of custom/customised prefabricated foot orthoses versus a control (sham orthoses) in improving health‐related quality of life after 3 months in children with juvenile idiopathic arthritis. This figure represents the child‐reported scores of the Physical functioning subscale Generic Module, Paediatric Quality of Life Inventory. CI, confidence interval; IV, inverse variance.

**FIGURE 10:**
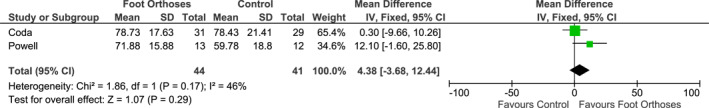
Meta‐analysis of the effectiveness of custom/customised prefabricated foot orthoses versus a control (sham orthoses) in improving health‐related quality of life after 3 months in children with juvenile idiopathic arthritis. This figure represents the parent‐reported scores of the Physical functioning subscale Generic Module, Paediatric Quality of Life Inventory. CI, confidence interval; IV, inverse variance.

Baydogan et al. (2015) did not provide means and standard deviations of outcome data and subsequently were unable to be included in statistical analysis [35]. All statistical analyses conducted for all single comparisons are presented in Table 4 (physical therapies) and Table 5 (mechanical therapies). Figures 2, 3, 4, 5, 6, 7, 8, 9, 10 present forest plot graphs for all the meta‐analyses performed.

**TABLE 4:** Statistical analysis and data for all standalone comparisons for physical therapy studies.

Outcome and follow‐up period	Study	Group	No.	Mean ± SD	Group	No.	Mean ± SD	Effect estimate mean difference (95%)
Pain (VAS), 6 Weeks	Eid 2016	EMG + standard physical therapy	18	6.88 ± 0.83	Standard physical therapy	18	6.94 ± 0.72	−0.06 [−0.57, 0.45]
Pain (VAS), 12 Weeks	Eid 2016	EMG + standard physical therapy	18	3.33 ± 0.97	Standard physical therapy	18	4.94 ± 0.72	−1.61 [−2.17, −1.05]
Pain (VAS), 1 Month	Elnaggar 2016	Resistive underwater exercises + interferential Current therapy	15	6.7 ± 0.9	Standard physical therapy	15	7.5 ± 0.8	−0.80 [−1.41, −0.19]
Pain (VAS), 3 Month	Elnaggar 2016	Resistive underwater exercises + interferential Current therapy	15	3.5 ± 2.2	Standard physical therapy	15	6.7 ± 0.6	−3.2 [−4.35, −2.05]
Functional capacity (6MWT–metres), 3 Month^a^	Elnaggar 2021	Core stability exercises + standard physical therapy	17	75.18 ± 41.24	Standard physical therapy	16	16.13 ± 14.25	59.05 [38.24, 79.86]
Pain (VAS), 3 Month	Azab 2022	Pilates + standard physical therapy	19	4.57 ± 1.17	Standard physical therapy	18	5.94 ± 1.11	−1.37 [−2.10, −0.64]
Functional ability (CHAQ), 3 Month	Azab 2022	Pilates + standard physical therapy	19	0.78 ± 0.39	Standard physical therapy	18	1.12 ± 0.40	−0.34 [−0.59, −0.09]
Quality of life (PedsQL), 3 Month	Azab 2022	Pilates + standard physical therapy	19	80.59 ± 3.96	Standard physical therapy	18	75.16 ± 6.55	5.43 [1.92, 8.94]
Functional capacity (6MWT–metres), 12 Weeks	Elnaggar 2022	Aqua plyometric exercise program	24	540.42 ± 84.16	Standard physical therapy	24	499.79 ± 74.32	40.63 [−4.29, 85.55]
Functional capacity (6MWT–metres), 6 weeks	Elnaggar 2024	Accommodating variable‐resistance training	29	550.79 ± 82.13	Standard physical therapy	29	506.59 ± 70.62	44.20 [4.78, 83.62]
Functional capacity (4 × 10mSRT–seconds), 6 weeks	Elnaggar 2024	Accommodating variable‐resistance training	29	9.14 ± 2.23	Standard physical therapy	29	10.80 ± 1.30	−1.66 [−2.60, −0.72]
Functional capacity (TUDS–seconds), 6 weeks	Elnaggar 2024	Accommodating variable‐resistance training	29	13.58 ± 3.92	Standard physical therapy	29	16.41 ± 3.75	−2.83 [−4.80, −0.86]

Abbreviations: 6MWT: 6‐min walk test; CHAQ: Childhood health assessment questionnaire; EMG: electromyographic feedback training; PedsQL: Paediatric quality of life inventory; SD: standard deviation; SRT: shuttle run test; TUDS: timed up and down stairs; VAS: Visual Analogue Scale.

^a^ Analysis for this outcome was conducted based on change from baseline means and standard deviations.

**TABLE 5:** Statistical analysis and data for all standalone comparisons for mechanical therapy studies.

Outcome and follow‐up period	Study	Group	No.	Mean ± SD	Group	No.	Mean ± SD	Effect estimate mean difference (95%)
Pain (VAS) child 3 Month	Powell 2005	Neoprene inserts and shoes	12	28.4 ± 28.8	Shoes	13	28.2 ± 20.1	0.20 [–19.42 to 19.82]
Pain (VAS) child 3 Month	Powell 2005	Custom orthoses and shoes	15	13.2 ± 13	Neoprene inserts and shoes	12	28.4 ± 28.8	−15.20 [–32.77 to 2.37]
FFI Act limitation 3 Month	Powell 2005	Custom orthoses and shoes	15	8.54 ± 11.06	Shoes	13	27.92 ± 27.89	−19.38 [–35.54 to −3.22]
FFI foot pain 3 Month	Powell 2005	Custom orthoses and shoes	15	18.35 ± 17.05	Shoes	13	37.54 ± 25.47	−19.19 [–35.50 to −2.88]
FFI disability 3 Month	Powell 2005	Custom orthoses and shoes	15	15.6 ± 13.51	Shoes	13	34.15 ± 26.35	−18.55 [–34.42 to −2.68]
FFI Act limitation 3 Month	Powell 2005	Neoprene inserts and shoes	12	19.96 ± 19.73	Shoes	13	27.92 ± 27.89	−7.96 [–26.79 to 10.87]
FFI foot pain 3 Month	Powell 2005	Neoprene inserts and shoes	12	30.46 ± 25.56	Shoes	13	37.54 ± 25.47	−7.08 [–27.10 to 12.94]
FFI disability 3 Month	Powell 2005	Neoprene inserts and shoes	12	29.98 ± 25.26	Shoes	13	34.15 ± 26.35	−4.17 [–24.40 to 16.06]
FFI Act limitation 3 Month	Powell 2005	Custom orthoses and shoes	15	8.54 ± 11.06	Neoprene inserts and shoes	12	19.96 ± 19.73	−11.42 [–23.91 to 1.07]
FFI foot pain 3 Month	Powell 2005	Custom orthoses and shoes	15	18.35 ± 17.05	Neoprene inserts and shoes	12	30.46 ± 25.56	−12.11 [–28.95 to 4.73]
FFI disability 3 Month	Powell 2005	Custom orthoses and shoes	15	15.6 ± 13.51	Neoprene inserts and shoes	12	29.98 ± 25.26	−14.38 [–30.22 to 1.46]
Timed walking 3 Month	Powell 2005	Custom orthoses and shoes	15	7.03 ± 1.12	Shoes	13	8.36 ± 2.44	−1.33 [–2.77 to 0.11]
Timed walking 3 Month	Powell 2005	Neoprene inserts and shoes	12	7.98 ± 1.3	Shoes	13	8.36 ± 2.44	−0.38 [–1.90 to 1.14]
Timed walking 3 Month	Powell 2005	Custom orthoses and shoes	15	7.03 ± 1.12	Neoprene inserts and shoes	12	7.98 ± 1.3	−0.95 [–1.88 to −0.02]
PedsQL (G‐PF) child 3 Month	Powell 2005	Neoprene inserts and shoes	10	55.94 ± 17.46	Shoes	12	59.78 ± 18.8	−3.84 [–19.01 to 11.33]
PedsQL (G‐PF) parent 3 Month	Powell 2005	Neoprene inserts and shoes	10	55.31 ± 15.8	Shoes	12	55.95 ± 13.97	−0.64 [–13.22 to 11.94]
PedsQL (G‐PF) child 3 Month	Powell 2005	Custom orthoses and shoes	13	71.88 ± 15.88	Neoprene inserts and shoes	10	55.94 ± 17.46	15.94 [2.10 to 29.78]
PedsQL (G‐PF) parent 3 Month	Powell 2005	Custom orthoses and shoes	14	64.96 ± 19.92	Neoprene inserts and shoes	10	55.31 ± 15.8	9.65 [–4.09 to 23.39]
PedsQL generic child 3 Month	Coda 2014	Customised orthoses	31	81.69 ± 11.89	Sham insoles	29	78.79 ± 21.59	2.90 [−6.00 to 11.80]
PedsQL generic parent 3 Month	Coda 2014	Customised orthoses	31	78.12 ± 15.66	Sham insoles	29	78.92 ± 22.2	−0.80 [−10.58 to 8.98]
PedsQL generic child 6 Month	Coda 2014	Customised orthoses	31	85.22 ± 12.66	Sham insoles	28	81.26 ± 20.03	3.96 [−4.69 to 12.61]
PedsQL generic parent 6 Month	Coda 2014	Customised orthoses	31	81.51 ± 13.61	Sham insoles	28	78.48 ± 21.84	3.03 [−6.37 to 12.43]
Pain (VAS) child 4 Week	Fellas 2021	Customised orthoses	32	26.81 ± 23.11	Sham insoles	30	40.97 ± 28.82	−14.16 [−27.22, −1.10]
Pain (VAS) child 6 Month	Fellas 2021	Customised orthoses	32	21.77 ± 21.41	Sham insoles	29	29.45 ± 23.33	−7.68 [−18.96, 3.60]
Pain (VAS) child 12 Month	Fellas 2021	Customised orthoses	29	29.11 ± 28.30	Sham insoles	27	37 ± 27.44	−7.89 [−22.49, 6.71]
Pain (VAS) parent 4 Week	Fellas 2021	Customised orthoses	32	25.27 ± 21.70	Sham insoles	30	33.93 ± 25.76	−8.66 [−20.56, 3.24]
Pain (VAS) parent 12 Month	Fellas 2021	Customised orthoses	29	25.11 ± 28.50	Sham insoles	27	28.52 ± 25.43	−3.41 [−17.54, 10.72]
PedsQL (RS) child 12 Month	Fellas 2021	Customised orthoses	29	71.76 ± 16.94	Sham insoles	27	69.89 ± 19.55	1.87 [−7.74, 11.48]
PedsQL (RS) parent 12 Month	Fellas 2021	Customised orthoses	29	69.32 ± 18.71	Sham insoles	27	72.77 ± 17.93	−3.45 [−13.05, 6.15]
JAFI‐impairment 3 Month	Fellas 2021	Customised orthoses	32	9.87 ± 5.38	Sham insoles	29	13.73 ± 8.09	−3.86 [−7.34, −0.38]
JAFI‐impairment 6 Month	Fellas 2021	Customised orthoses	32	9.23 ± 6.28	Sham insoles	29	12.52 ± 6.29	−3.29 [−3.29, −0.13]
JAFI‐impairment 12 Month	Fellas 2021	Customised orthoses	29	11.96 ± 7.21	Sham insoles	27	11.64 ± 7.42	0.32 [−3.52, 4.16]
JAFI‐Act limit 3 Month	Fellas 2021	Customised orthoses	32	8.77 ± 8.97	Sham insoles	29	13.73 ± 10.31	−4.96 [−9.83, −0.09]
JAFI‐Act limit 6 Month	Fellas 2021	Customised orthoses	32	7.71 ± 6.86	Sham insoles	29	12.62 ± 8.37	−4.91 [−8.77, −1.05]
JAFI‐Act limit 12 Month	Fellas 2021	Customised orthoses	29	10.81 ± 9.54	Sham insoles	27	12.46 ± 10.46	−1.65 [−6.91, 3.61]
JAFI‐participation 3 Month	Fellas 2021	Customised orthoses	32	3.42 ± 2.90	Sham insoles	29	5.23 ± 4.14	−1.81 [−3.62, 0.00]
JAFI‐participation 6 Month	Fellas 2021	Customised orthoses	32	3.19 ± 2.43	Sham insoles	29	4.17 ± 3.00	−0.98 [−2.36, 0.40]
JAFI‐participation 12 Month	Fellas 2021	Customised orthoses	29	4.11 ± 3.73	Sham insoles	27	5.25 ± 3.47	−1.14 [−3.03, 0.75]
Swollen joints 6 Month	Fellas 2021	Customised orthoses	32	0.47 ± 0.98	Sham insoles	29	1.07 ± 1.69	−0.60 [−1.30, 0.10]
Tender joints 6 Month	Fellas 2021	Customised orthoses	32	1.19 ± 2.43	Sham insoles	29	2.41 ± 2.63	−1.22 [−2.49, 0.05]
Cadence 3 Month	Fellas 2022	Customised orthoses	32	120.88 ± 21.06	Sham insoles	29	124.65 ± 23.59	−3.77 [−15.04, 7.5]
Cadence 6 Month	Fellas 2022	Customised orthoses	32	115.66 ± 21.98	Sham insoles	29	120.28 ± 21.6	−4.62 [−15.57, 6.33]
Stance time 3 Month	Fellas 2022	Customised orthoses	32	1.25 ± 0.23	Sham insoles	29	1.21 ± 0.26	0.04 [−0.08, 0.16]
Stance time 6 Month	Fellas 2022	Customised orthoses	32	1.26 ± 0.26	Sham insoles	29	1.22 ± 0.28	0.04 [−0.10, 0.18]
Swing time 3 Month	Fellas 2022	Customised orthoses	32	0.82 ± 0.13	Sham insoles	29	0.81 ± 0.14	0.02 [−0.05, 0.09]
Swing time 6 Month	Fellas 2022	Customised orthoses	32	0.85 ± 0.16	Sham insoles	29	0.85 ± 0.13	0 [−0.07, 0.07]

Abbreviations: FFI: Foot Function Index; G‐PF: Generic—physical functioning; JAFI: Juvenile Arthritis Foot and Ankle Disability Index; PedsQL: Paediatric quality of life inventory; RS: rheumatology score; SD: standard deviation; VAS: Visual Analogue Scale.

## 4 Discussion

In this review a total of 452 participants with JIA were included and 67.7% of them were female. Ten interventions (six physical and four mechanical) were examined for their efficacy on lower limb problems with the most common clinical problem being pain. These clinical trials were conducted across five countries including Saudi Arabia, Australia, Turkey, The United States of America and The United Kingdom.

### 4.1 Physical Therapies

The original version of this systematic review was conducted and completed in 2015 [12]. In the 2015 publication, no RCT evaluated physical therapy, however, since 2015, six RCTs have explored the effect of physical therapies on lower limb problems in children with JIA. Below each physical therapy will be discussed on its efficacy compared to the control for the outcomes of interest.

#### 4.1.1 Electromyographic Biofeedback Training

Electromyographic (EMG) biofeedback training when combined with standard physical therapy versus standard physical therapy alone was not statistically significant in the reduction of lower limb pain after 6 weeks (−0.06 [–0.57, 0.45]).

#### 4.1.2 Resistive Underwater Exercises and Interferential Current Therapy

Resistive underwater exercises and interferential current therapy versus standard therapy alone was statistically significant in reducing lower limb pain after one (−0.80 [−1.41, −0.19]) and 3 months (−3.2 [−4.35, −2.05]). The recommended minimal important clinical difference for pain in children with JIA is approximately 8 mm [47]. Therefore, both one‐ and 3‐month follow‐ups were clinically significant in favour of the trial group with a much greater clinical effect seen after 3 months.

#### 4.1.3 Core Stability Exercises

Elnaggar et al. (2021) used the six‐minute walk test to assess the effect of core stability exercises have on functional capacity. The baseline scores of this outcome were significantly different and therefore authors were requested to provide individual participant data so means and standard deviations of change in baseline to follow‐up scores could be calculated. The change from baseline analysis showed that core stability exercises with standard physical therapy was statistically more significant (59.05 [38.24 to 79.86]) than standard physical therapy alone in the improvement of functional capacity in children with polyarticular JIA after 3 months of intervention. This effect fell just short of clinical significance according to the recommended minimal important clinical difference of 65.1 for the six‐minute walk test in children with JIA [48].

#### 4.1.4 Pilates

Pilates combined with standard physical therapy versus standard physical therapy alone was statistically and clinically significant in the reduction of pain after 3 months follow‐up (−1.37 [−2.10, −0.64]). Functional ability (−0.34 [−0.59, −0.09]) and quality of life (5.43 [1.92, 8.94]) assessed by the Childhood Health Assessment Questionnaire (CHAQ) and PedsQL respectively were both statistically and clinically significant in favour of Pilates group. The minimal important clinical difference for CHAQ in children with JIA is a change in score of 0.13 with a lower score more desirable outcome [49] and PedsQL a change of 5 between follow‐ups with a higher score a more desirable outcome [21].

#### 4.1.5 Aqua Plyometric Exercise Program

Functional capacity was measured using the 6‐min walk test. An aqua plyometric exercise program versus standard physical was not statistically significant in improving functional capacity (40.63 [−4.29, 85.55]) after 12 weeks of intervention.

#### 4.1.6 Proprioceptive and Balance Exercise Program

Statistical analysis was unable to be conducted for this intervention as authors of the study did not provide means and standard deviations for the outcomes of interest. Authors reported data as not normally distributed and used non‐parametric analyses. After 12 weeks of intervention authors reported no statistically significant reductions in pain between groups when assessed at rest but pain was significantly reduced both statistically and clinically when assessed after exercise in favour of the proprioceptive and balance exercise program group. Other relevant outcomes that were statistically significant in favour of the trial group were the 10‐m walk test (*p* = 0.016) and 10‐stair climbing test (*p* = 0.011), however it is unclear if these values are clinically significant. Lastly, the CHAQ was used to measure functional ability which was not statistically or clinically significant after 12 weeks of observation (*p* = 0.708).

#### 4.1.7 Accommodating Variable‐Resistance Training Program

Functional capacity was measured using the 6‐min walk test, shuttle run test and timed up and down stairs test. After 6 weeks of intervention, participants who completed the accommodating variable‐resistance training program displayed statistically significant improvements versus the control in all three measures of functional capacity (6‐min walk test, 44.20 [4.78, 83.62]; shuttle run, −1.66 [−2.60, −0.72], timed up and down stairs, −2.83 [−4.80, −0.86]). The clinical implications of these results are unclear.

### 4.2 Mechanical Therapies

Foot orthoses, custom or customised, remain the only type of mechanical therapy explored in RCTs to mitigate lower limb problems in children with JIA. Below the outcomes assessed in these clinical trials have been subdivided and discussed.

#### 4.2.1 Custom/Customised Foot Orthoses

##### 4.2.1.1 Pain

Powell et al. (2005), Coda et al. (2014) and Fellas et al. (2021) explored foot and ankle pain as the primary outcome in their respective studies. Pain was child‐reported only in Powell et al. (2005), parent‐reported only in Coda et al. (2014) and Fellas et al. (2021) collected both child and parent reported pain outcomes. Therefore, a meta‐analysis of child‐reported pain at 3 months post intervention was conducted between Fellas et al. (2021) and Powell et al. (2005), then meta‐analyses of parent‐reported pain at 3 and 6 months between Fellas et al. (2021) and Coda et al. (2014). Results of the meta‐analyses showed that after 3 months of intervention, custom or customised foot orthoses were statistically and clinically more significant in the reduction of foot and ankle pain verses the control in children with JIA. This was for both child (−21.45 [−30.18 to −12.73]) and parent (−11.08 [−20.25 to −1.90]) reported pain. This effect was not extended to the meta‐analysis at 6 months (−6.74 [−14.57 to 1.08], however, analysis indicated a strong trend in favour of customised foot orthoses.

Single study analysis for child‐reported pain showed that customised foot orthoses were statistically and clinically more effective in reducing pain vs. a sham device at 4‐week (−14.16 [−27.22 to −1.10]); however, customised were not more effective than the control when pain was assessed at 6 (−7.68 [−18.96 to 3.60]) and 12 months (−7.89 [−22.49 to 6.71]) post intervention. Lastly, parent‐reported pain was not statistically significant at 4‐week (−8.66 [−20.56 to 3.24]) and 12 months (−8.66 [−20.56 to 3.24]) post intervention when comparing customised foot orthoses to a sham device.

##### 4.2.1.2 Quality of Life

Powell et al. (2005), Coda et al. (2014) and Fellas et al. (2021) used the PedsQL to assess the impact of foot orthoses on quality of life. Powell et al. (2005) only used the physical functioning subscale of the PedsQL Generic Module, while Coda et al. (2014) assessed quality of life with both the Generic and rheumatology modules but only published the rheumatology module [13]. Fellas et al. (2021) used only the PedsQL rheumatology module in their study.

Meta‐analyses of the physical functioning scale of the Generic PedsQL module at 3 months for both child (1.77 [−6.35 to 9.90]) and parent (4.38 [−3.68, 12.44]) reports were not statistically significant in favour of the trial group. Meta‐analysis of the child‐reported pediatric rheumatology PedsQL module between Coda et al. (2014) and Fellas et al. (2021) showed that customised prefabricated foot orthoses were statistically significant (6.28 [0.21 to 12.34]) in improving quality of life 3 months post intervention versus sham orthoses. This was also clinically significant as the average mean difference was above 5 which is the recommended minimal important clinical difference between intervals for the PedsQL in children with JIA [21]. However, customised foot orthoses were not statistically significant for the pediatric rheumatology module for child‐reported at 6 months, and parent‐reported at 3‐ and 6‐month follow‐up.

Single study analysis for the Generic PedsQL module showed that customised foot orthoses were not statistically significant in improving quality of life for both child and parent reports at 3‐ and 6‐month follow‐up versus the control group. This was also the case when the pediatric rheumatology module was assessed by child and parent at 12‐month post intervention.

##### 4.2.1.3 Foot Function

Powell et al. (2005) assessed foot function using the foot function index. Custom foot orthoses were statistically and clinically more significant in improving all domains of the foot function index versus the shoes only group at 3 months. Both custom foot orthoses compared to neoprene flat inserts and neoprene flat inserts versus shoes only group were not statistically significant in improving any of the foot function index domains at 3 months follow‐up.

##### 4.2.1.4 Timed Walking

At 3‐month follow‐up, mean walking speed was significantly faster in children with custom foot orthoses than in those with neoprene inserts (−0.95 [–1.88 to −0.02]). The minimal important clinical difference is unknown, and it is possible that the difference is too small to be clinically important. No significant differences in walking speed were found between children with custom Foot orthoses and shoes versus shoes alone or neoprene inserts and shoes versus shoes alone.

##### 4.2.1.5 Foot Disability

Fellas et al. (2021) assessed foot disability using the juvenile arthritis foot ankle disability index (JAFI) which is validated in children with JIA [20]. This outcome was assessed at 3‐, 6‐ and 12‐month follow‐ups. The JAFI is calculated and analysed into three domains: impairment, activity limitation and participation restriction. Customised foot orthoses were statistically significant in reducing foot disability versus the control group (sham foot orthoses) for the impairment domain at 3‐ (−3.86 [−7.34 to −0.38]) and 6‐ months (−3.29 [−3.29 to −0.13]), as well as the activity limitation domain at 3‐ (−4.96 [−9.83 to −0.09]) and 6‐month (−4.91 [−8.77 to −1.05]) follow‐up. The minimal important clinical difference is unknown and therefore is it unclear whether the mean difference effects were clinically perceivable by participants. Statistical significance was not reached for any JAFI domain at 12‐month and any of the participation restriction follow‐ups.

##### 4.2.1.6 Joint Involvement

Fellas et al. (2021) assessed the impact of their intervention on lower limb joint swelling and tenderness. Joint assessments were conducted by pediatric rheumatologists who were blinded to what group participants were in. Analysis of the total joint swelling and tenderness count from baseline to 6‐month showed that customised foot orthoses versus sham orthoses were not statistically significant in reducing swollen (−0.60 [−1.30 to 0.10]) or tender (−1.22 [−2.49 to 0.05]) lower limb joints in children with JIA.

##### 4.2.1.7 Gait Parameters

Cadence, stance and swing time are measures of walking efficiency and were captured by Fellas et al. (2022). Analysis results showed that customised foot orthoses were not statistically significant in improving cadence, stance or swing time at 3‐ and 6‐ months follow‐up versus sham orthoses.

### 4.3 Recommendations for Clinical Practice

When interpreting the results of this systematic review, it is important clinicians should be aware of that only three of the 11 included studies were single‐blinded (blinding the participants, and never the person administering the intervention). The remaining included studies did not blind participants or the person administering the intervention. This area was identified as being at high risk of bias and results should be interpreted with care.

Evidence from this systematic review indicates that if a patient with JIA presents with lower limb pain, then a resistive underwater and interferential current therapy program or Pilates may be implemented as part of their rehabilitation and ongoing management. The sustained reduction of pain with these physical therapies after 3 months is however unclear. Electromyographic biofeedback training and balance proprioceptive training may be used as more of a holistic approach in reducing pain up to 3 months. It is generally unclear if functional capacity would be significantly improved with the addition of core stability exercises, an aqua plyometric exercise program or a proprioceptive and balance exercise program. Accommodating variable‐resistance training was statistically significant in improving functional capacity, however, the clinical significance of these findings are unclear.

Custom or customised foot orthoses can be prescribed for children and adolescents with JIA to significantly reduce foot and ankle pain, particularly in the first 3 months of intervention. Sustained efficacy of pain reduction should be closely monitored by clinicians to ensure the foot orthoses are providing a clinical benefit to the patient. Clinicians should also consider the evidence of foot orthoses have on other outcomes such as foot function, foot and ankle disability and overall quality of life which appears to be limited any time after 3 months's follow‐up. Another important factor to consider when prescribing foot orthoses is whether to prescribe custom or customised devices. Custom foot orthoses are significantly more expensive than customised prefabricated foot orthoses for the patient to acquire from a private clinic. Customised prefabricated foot orthoses can be prescribed on the same day of the initial biomechanical consultation without compromising clinical effectiveness and typically with less cost to the patient. JIA patients may also sustain changes to their lower limb which may require a change in prescription or fundamental design of the orthoses. Moreover, pediatric patient's feet will grow and require frequent replacing. Lastly, it is important for clinicians to consider if custom or customised foot orthoses are prescribed, then prolonged use of this intervention should be monitored carefully for its viability in producing ongoing improvements in clinical outcomes.

### 4.4 Limitations

Some limitations to this systematic review should be considered. First this review only included RCTs that explored the effect of interventions on the lower limb. Second, only pre‐specified outcomes were analysed based on our published protocol [18]. Therefore, some of the outcomes explored by included studies were not included in the statistical analysis and discussion of this review.

#### 4.4.1 Limitations of Included Studies

The first limitation of included studies to highlight was the lack of blinding that occurred. Only three of the eleven studies included in this review were single‐blinded RCTs in which none of the physical therapy studies were blinded. Double blinding non‐pharmacological RCTs can be difficult for research teams to achieve due to the nature of delivering a blinded intervention to participants. However, it is recommended that a minimum of single blinding is carried out for these studies to minimise the risk of bias in the results [50]. At least five of the RCTs sample size calculations produced seemingly low participant numbers and it was unclear if these numbers were sufficient to detect true change. Another limitation of the included studies was 68% (347/510) of JIA participants were diagnosed with polyarticular subtype. The high percentage of polyarticular JIA participants was due to six of the eleven studies (Eid et al. 2016; Elnaggar et al. 2016; Elnaggar et al. 2021; Elnaggar et al. 2022; Azab et al. 2022; Elnaggar et al. 2024) only including this subtype ultimately affecting the generalisability of findings.

### 4.5 Recommendations for Future Research

Currently, there are no RCTs that explore the effect of physical therapies on lower limb problems in children with JIA beyond 3 months. Since JIA is a chronic condition, it is recommended for future researchers to design their clinical trials with longer observational periods to determine if physical therapies have sustained efficacy. It is also important to consider the implications of participants with JIA encountering a disease flare during a non‐pharmacological RCT due to increased symptoms and likely significant changes in medication which have a high chance of confounding data. Therefore, future researchers in physical and mechanical therapies should closely monitor whether their participants experience disease flare in RCTs longer than 3 months and account for this in their analyses. Custom or customised foot orthoses remain the only mechanical therapy focusing on the lower limb in children with JIA. Future research could explore the effect of different types of foot orthoses in the same trial to determine what is the most accepted and cost‐effective for children with JIA. Other mechanical therapies that may be useful to test in combination with Foot orthoses are taping or bracing. Lastly, authors who may conduct future clinical trials are recommended to blind participants to their intervention as feasible as possible as well as blinding outcome assessors and research personnel.

## 5 Conclusion

Based on meta‐analysis, custom or customised foot orthoses are statistically and clinically significant in reducing foot and ankle pain in children with JIA in the first 3 months of intervention. This effect did not extend past 3 months despite statistical and clinical trends suggesting prolonged pain reduction in favour of customised foot orthoses. There were inconsistent trends in the effect of custom or customised foot orthoses on quality of life, foot and ankle disability and foot function. Physical therapies such as electromyographic biofeedback training, Pilates and resistive underwater and interferential current therapy were statistically and clinically significant in reducing lower limb pain after 3 months of intervention versus standard physical therapy. Pilates was also effective in improving quality of life and reducing disability (CHAQ). Most studies included in this systematic review were subject to a high risk of reporting bias as there was limited attempt to blind research personnel and outcome assessors to participant intervention groups. Approximately half of the included studies were hampered by small sample sizes and a homogenous cohort of polyarticular JIA participants. Overall, further research is required, particularly physical therapy based RCTs that are at least single blinded, with longer follow‐ups and larger sample sizes to provide more conclusive evidence on the effect of physical therapies in improve lower limb problems in children with JIA.

## Author Contributions

**Antoni Fellas:** conceptualisation, data curation, methodology, formal analysis, project administration, validation, writing – original draft, writing – review and editing. **Fiona Hawke:** conceptualisation, methodology, formal analysis, supervision, writing – original draft, writing – review and editing. **Mohammed Maarj:** data curation, methodology, writing – original draft, writing – review and editing. **Davinder Singh‐Grewal:** conceptualisation, methodology, supervision, writing – original draft, writing – review and editing. **Derek Santos:** conceptualisation, methodology, supervision, writing – original draft, writing – review and editing. **Andrea Coda:** conceptualisation, methodology, formal analysis, supervision, writing – original draft, writing – review and editing.

## Funding

The authors have nothing to report.

## Conflicts of Interest

Dr Fellas, Dr Coda, Prof Santos and A/Prof Singh‐Grewal may be authors of papers that are included in this systematic review.

## Data Availability

The data that support the findings of this study are available from the corresponding author upon reasonable request.
